# Determining the prevalence of high-risk human papillomavirus infection using a novel cervical precancer screening approach in incarcerated women at the Nsawam Medium Security Prison, Ghana

**DOI:** 10.3332/ecancer.2021.1248

**Published:** 2021-06-14

**Authors:** Lawrence Kofi Acheampong, Kofi Effah, Joseph Emmanuel Amuah, Ethel Tekpor, Comfort Mawusi Wormenor, Isaac Gedzah, Seyram Kemawor, Ateba Cynthia Kachana, Peace Afi Danso, Nana Owusu Mensah Essel, Mabel Asomaning, Dominic Agyiri, Patrick Kafui Akakpo

**Affiliations:** 1 Ghana Prisons Service, Nsawam Medium Security Prison, Prisons Hospital, PO Box 305, Nsawam, Eastern Region, Ghana; 2 Catholic Hospital, Battor, Ghana; 3 Lotus Medical Group, PO Box SE 1698, Kumasi, Ghana; 4 Ghana Prisons Service, Ghana Prisons Headquarters, PO Box 129, Accra, Ghana; 5 Department of Pathology, School of Medical Sciences, University of Cape Coast, Cape Coast Teaching Hospital, Cape Coast, Ghana

**Keywords:** prisoners, cancer, screening, Ghana

## Abstract

**Background:**

Across Ghana, females comprise 1.2% of the entire prison population (*n* = 15,463). Cervical cancer screening services are however nonexistent and the prevalence of high-risk human papillomavirus (hr-HPV) and cervical precancer is undocumented. We aimed to screen and treat inmates for cervical precancer and determine the prevalence of hr-HPV using the novel AmpFire HPV detection system combined with colposcopy by trained nurses using a mobile colposcope (the Enhanced Visual Assessment (EVA) system).

**Methods:**

A descriptive cross-sectional study design was employed, involving all incarcerated women at the Nsawam Medium Security Prison, Ghana. After counselling and informed consent, women underwent a structured questionnaire-based interview entered into a Microsoft Excel spreadsheet. Women were co-tested for cervical pre-cancer and hr-HPV by two trained nurses using dry brush cervical samples for 15 hr-HPV types (AmpFire HPV test) after which mobile colposcopy with the EVA system was performed. EVA images were reviewed by a gynaecologist. Frequencies and percentages were used to describe categorical data, while means, standard deviations, medians and interquartile ranges (IQRs) were used to describe continuous data.

**Results:**

75% of the women were convicts with a median sentence of 5 years (IQR: 2–10 years). Their mean age was 41.1 years (standard deviation: 15.5 years, range: 19–97 years). The self-reported prevalence rate of HIV was 13.1% (95% confidence interval (CI): 7.5%–21.9%), all of whom were receiving treatment. The hr-HPV prevalence rate was 47.6% (CI: 36.9%–58.3%) in the general population of imprisoned women and 63.6% (CI: 35.4%–84.8%) among HIV positive women. Six percent (6%) had lesions on the cervix, of which 3.6% were treated with thermal coagulation and 2.4% were treated with loop electrosurgical excision procedure. The average age of hr-HPV positive women was 37.8 years.

**Conclusions:**

There is a high prevalence of hr-HPV infection among women in custody at the Nsawam Medium Security Prison. These women will benefit from structured cervical cancer prevention services, including treatment for abnormalities that are picked up during such screening.

## Introduction

Cervical cancer was the leading cause of cancer-related death in women in Sub-Saharan Africa in a 2018 report [1]. Worldwide, however, cervical cancer was the fourth most common cancer in women. There is a wide variation in the estimated age-standardised incidence of cervical cancer among various countries, with rates ranging from less than 2 to 75 per 100,000 women [1]. The regions of the world with the higher reported incidence rates invariably have the highest cervical cancer related mortality [1]. The differential epidemiology of cervical cancer in the world is attributable to availability and access to cervical cancer prevention strategies [2].

Knowledge that infection with high-risk Human Papilloma Virus (hr-HPV) is a necessary cause of nearly all cervical cancers has resulted in major changes in cervical cancer prevention methods [3]. There is now the possibility of vaccinating girls against the virus in addition to cervical cancer screening at a later age. The difference in screening strategy between the past and present is that primary hr-HPV screening is now more acceptable than Pap smear and other methods that depend on visualisation of the cervix. Primary hr-HPV screening though routine in most high-income countries is however not readily available in low-middIe income countries (LMICs) such as Ghana. Ironically, HPV infection rates are highest in these LMICs and these same countries lack HPV vaccination programmes. In Ghana, prevalence of HPV 16/18 among women with normal cytology is 4.3%, among women with low-grade cervical lesions [low-grade squamous intraepithelial lesion (LSIL)/cervical intraepithelial neoplasia I (CIN I)], it is 24.3%, among women with high-grade cervical lesions [high-grade squamous intraepithelial lesion (HSIL)/cervical intraepithelial neoplasia II & III (CIN II & III)/carcinoma in situ (CIS)] it is 35.6%, and among women with cervical cancer, it is 59.2% [4]. Where HPV vaccination and screening are available, women are unable to access the service due to cost or to unfavourable location of the service precluding access [5].

In LMICs such as Ghana, it has been suggested that a mix of innovative screening methods tailored to the patient’s unique circumstances need to be employed for cervical cancer screening, if any gains are to be made in the fight to reduce the burden of cervical cancer [2, 5]. Improving accessibility to cervical cancer screening is crucial to reducing the burden of cervical cancer in Ghana considering that there are reported disparities between various regions in the country [6].

It has been reported that imprisoned women are at increased risk of cervical cancer but are also less likely to have been screened for cervical cancer [7]. These women report multiple risk factors for HPV and cervical cancer, and have up to a fivefold increased risk of cervical cancer when compared with women who are not incarcerated [8]. As at September 2019, it was estimated that Ghana had a prison population rate of close to 50 per 100,000 population, with females comprising 1.2% of this number, a total of 15,463 [9]. Ghana ranks 56th in terms of prison overcrowding worldwide, with an occupancy level of 155.5% [9]. Although some prisons in Ghana have clinics, these facilities are often poorly resourced and thus do not offer added screening services which generally are not considered essential.

There is a paucity of data in HPV testing among incarcerated women in low resource countries. Most studies describing the epidemiology of hr-HPV infection among incarcerated women have emanated from relatively higher-income settings [10–16]. In lower income settings, studies have been done using non-routine clinical approaches [10, 12, 14, 15]. Few studies have been done in low resource settings using the AmpFire HPV detection system and these have also been in the context of research or in a one-time programme, not using a routine clinical approach in a low resource country. In our work, we used the routine clinical screening approach at Catholic Hospital, Battor. The AmpFire HPV detection system has been used for clinical screening in Battor since June 2019, when it became the preferred choice over careHPV which had been in use since June 2016. This is because the AmpFire assay has the advantage of some additional genotyping. Colposcopy performed by trained nurses using the mobile colposcope (the Enhanced Visual Assessment (EVA) system) has been used for clinical screening in Battor since August 2016. This study, therefore, gives an idea about what to expect when incarcerated women are included in routine cervical cancer screening programmes in Ghana. While awaiting a national programme on cervical cancer prevention, there is the need, when the opportunity is presented, to ensure that women in prisons are provided with oncologic screening services, particularly regarding cancers with infectious cause. One such rare opportunity arose on 27 and 28 September 2019 when the staff of the Cervical Cancer Prevention and Training Centre, Battor, Ghana provided cervical screening services to women at Nsawam Prisons, one of seven female prisons in the country and the most populated.

We aimed to screen and treat incarcerated women for cervical precancer and determine the prevalence of hr-HPV using the novel AmpFire HPV detection system. This is in line with a previous study that showed that the sensitivity for CIN2 or worse was similar between AmpFire and Cobas tests. The sensitivity for AmpFire ranged between 95.74% and 96.81% compared to that of Cobas which ranged from 92.55% to 95.74%. Specificity was significantly better for AmpFire (ranged from 89.8% to 90.8) than Cobas (88.5% to 91%) [17]. This work provides the first results of prevalence and distribution of hr-HPV among incarcerated women in Ghana. We hope that the results will influence policy on screening and preventing cervical cancer to bridge the gap in equality.

## Materials and methods

### Study design and sample

**Design**: We conducted a descriptive cross-sectional study involving the screening of 85 incarcerated women at the Nsawam Medium Security Prison in Ghana on 27–28 September 2019 to assess the prevalence of hr-HPV among them. At the time of the study, 90 women were incarcerated at the prison and 85 of them agreed to participate in the study.

**Setting and participants**: All incarcerated women at the Nsawam Medium Security Prison, Ghana on 27–28 September 2019.

After counselling and informed consent, women underwent a structured interview based on a questionnaire and their responses were recorded directly into a Microsoft Excel spreadsheet database developed specifically for recording all data associated with cervical screening and treatment. Both the questionnaire and electronic data capture tool were developed at the Cervical Cancer Prevention and Training Centre (CCPTC) at the Catholic Hospital, Battor. These tools have been in use since 2017 for capturing all data on cervical screening and treatment conducted by the team from CCPTC.

To ensure the utmost privacy of clients, all clients were identified by unique alphanumeric identifiers and none of their personal identification data such as names and previous addresses were obtained during the screening.

### Sample collection

For all the women, a speculum was inserted in the vagina to expose the cervix and samples were collected from the cervix with a sterile brush which was put in a collection tube and submitted to the laboratory technologists for testing.

### Ethical considerations

Ethical clearance was obtained from the Ethical Review Committee of the Ghana Prison Service. All women whose images were used in the manuscript gave us consent to use the images (SPO.1088/20/993^J^).

### Variables

#### HPV testing with the AmpFire HPV detection system

HPV testing was performed using the AmpFire HPV detection technology (Atila BioSystems, Mountain View, CA, USA). It is an isothermal polymerase chain reaction (PCR) assay that uses minimum instrumentation and detects 15 hr-HPV (16, 18, 31, 33, 35, 39, 45, 51, 52, 53, 56, 58, 59, 66, 68) in a single tube reaction and simultaneously identifies specifically the presence of types 16 and 18. Dry brush samples were submitted immediately at room temperature to the laboratory for testing. Samples could be processed individually or batched (1 to 94 samples per run). DNA extraction was not required and sample processing to final results took less than 2 hours [17].

The AmpFire HPV screen kit contained the reaction mix (with buffer, enzymes and deoxyribonucleoside triphosphate), primer mix (with primers and probes), positive control and negative control. Twelve microlitres of reaction mix was mixed with 11 µL of primer mix in a 0.2 mL reaction single tube, 8-well strip, or 96-well plate. One millilitre of lyse buffer was added to the dry sample brush in a 5 mL empty tube and left to sit at room temperature for 20 minutes after it had been vortexed. After this, 2 µL processed samples were added to the reaction tube to bring the total volume to 25 µL. The reaction tubes were incubated at 60°C for 60 minutes in the Atila Power 96 real-time PCR system (the PCR program is set with denaturation step at 60°C for 30 seconds, followed by extension step at 60°C for 30 seconds while taking fluorescence reading. A total of 60 cycles were run with fluorescence recorded once per minute from the FAM/HEX/ROX/CY5 channels. The results of the Ct values for each amplification curve in all fluorescence channels were automatically reported by the thermocycling software system. For each sample, an exponential amplification curve in the CY5, ROX, FAM and HEX channels indicated the presence of the DNA of HPV16, HPV 18, non-16/18 hr-HPV types and internal control, respectively [17]. The lack of exponential amplification curve in the HEX channel was interpreted as an invalid result. The negative and positive controls were included in each assay to ensure the quality of the assay and avoid possible contamination.

#### Mobile colposcopy with the EVA system

After passing a speculum and taking a sample with a dry brush for HPV DNA testing, each of the two trained nurses, working in different rooms, independently performed colposcopy with a mobile colposcope, theEVA system (MobileODT, Tel Aviv, Israel). The EVA System consists of a mobile colposcope built around a smartphone, and an online image portal for storing images. A smartphone app is used to control the mobile colposcope, and upload pictures to the image portal or to store images on the phone for review [18]. The adequacy of colposcopy, the transformation zone (TZ) type and any lesions found on the cervix or in the vagina were recorded by the nurses. The images were anonymised by assigning them special codes. A gynaecologist subsequently reviewed the images.

### Key definitions

#### Transformation zone types

Type 1 (TZ1): The entire circumference of squamocolumnar junction is visible; fully ectocervical.

Type 2 (TZ2): The entire circumference of squamocolumnar junction is visible; partly or fully endocervical.

Type 3 (TZ3): The entire circumference of the squamocolumnar junction is not visible; partly or fully endocervical.

### Bias

Self-reported data such as HIV status may be under-estimated since we did not conduct HIV testing as part of the screening exercise. This could lead to an over-estimate of the HPV prevalence among HIV positive incarcerated women.

### Statistical analysis

Percentages were calculated for categorical data and means, and standard deviations were used to describe continuous data. Skewed continuous data (such as period of incarceration) were summarised using medians and interquartile ranges (IQRs). For our primary objective of estimating the prevalence of hr-HPV among incarcerated women, we used simple percentages and reported 95% confidence intervals (CIs). For the secondary objectives, we explored the association between age of incarcerated women and period of incarceration and hr-HPV positivity using grouped box plots. In addition, we compared the period of incarceration between those with hr-HPV positivity and those without using a Wilcoxon rank-sum test. This was done because the period of incarceration was highly right skewed. A student’s *t*-test was used to compare the means of two populations in order to explore further the relationship between hr-HPV positivity and the age of incarcerated women.

We reported 95% CIs for all key estimates obtained. All statistical analyses were performed using STATA version 15 (StataCorp LLC, College Station, Texas, USA). We considered a statistical significance level of 5% and reported all *p*-values for all our hypothesis tests.

## Results

Informed consent was sought directly from all 90 incarcerated women at this prison for cervical screening.

**Figure 1.**
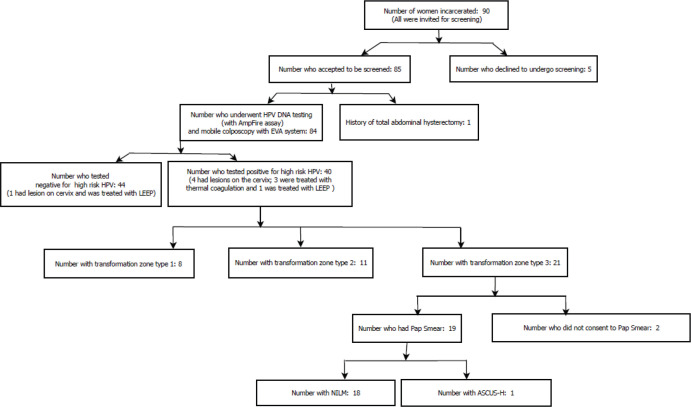
Overview of screening and treatment of imprisoned women of Nsawam Medium Security Prisons, Ghana.

Five of the women did not consent to be screened for the following reasons: Two of them had undergone total abdominal hysterectomy and so did not have a cervix, one said she was too old and did not expect to live much longer and so did not need cervical screening, one had recently undergone cervical screening prior to our visit and one was uncomfortable with strangers observing her genitalia. Of the 85 women who agreed to be screened, 1 of them did not have a cervix (due to a previous total abdominal hysterectomy); thus, 84 of the women were screened. Figure 1 provides an overview of the screening process.

Majority (75%, 63/84) of the women were convicts with a median sentence of 5 years (IQR: 2–10 years). Those on remand comprised 14.3% (12 women), 6% (5 women) were condemned and the rest (4.8%) had been sentenced to life imprisonment.

The median period of incarceration as at the time of screening of the women was about 1 year (IQR: 0.5–2.5 years) and 13.1% had been in jail for at least 5 years. The mean age of the participants was 41.1 years (standard deviation: 15.5, range: 19–97). In terms of marital status, 25% of the women were married, 22.6% were divorced, 19.1% were single and 17.9% were widowed. The remaining women were in some form of steady relationship with a partner. The highest education level was varied with 14.3% having attained tertiary education, 52.4% having attained secondary education and 15.5%

having attained elementary education. The rest (17.8%) had had no formal education. An overwhelming majority (84.5%) were Christian, 13.10% were Muslim and the rest (2.4%) were practitioners of African traditional religion. None of them earned an income.

About 11% were current or past smokers. Most of the women had had multiple births in the past, with 17.9% having had no children, 21.4% having had one child, 14.3% having had two children and the remaining 46.4% having had at least three children.

Fourteen percent (12/84) reported having undergone cervical screening in the past, most (9/12) of them having been screened only once (either by Pap or visual inspection with acetic acid (VIA)). None of them had been treated in the past following cervical screening. One had been vaccinated for HPV in the past.

The self-reported prevalence rate of HIV among the women was13.1% (95% CI: 7.5%–21.9%) and all the HIV positive incarcerated women were receiving treatment for that.

Of the 84 screened, 40 had hr-HPV infections, resulting in a hr-HPV prevalence rate of 47.6% (95% CI: 36.9%–58.3%). Of the 40 women infected with hr-HPV, 4 were infected with HPV 16 alone, 8 with HPV 18 alone and 23 with hr-HPV types other than HPV 16 and HPV 18. Some patients were infected with multiple hr-HPV types. Two women had HPV 16 and other hr-HPV types and three had HPV 18 and other hr-HPV types.

The positivity rate of hr-HPV infection among the HIV positive women was 63.6% (95% CI: 35.4%–84.8%).

Colposcopy using the EVA system was adequate for all 84 incarcerated women; 13.1% had TZ1, 24.1% had TZ2 and 65.5% had TZ3. Five (6%) had lesions on the cervix (aceto-whitening). Three were treated with thermal coagulation and two with loop electrosurgical excision procedure (LEEP).

**Figure 2.**
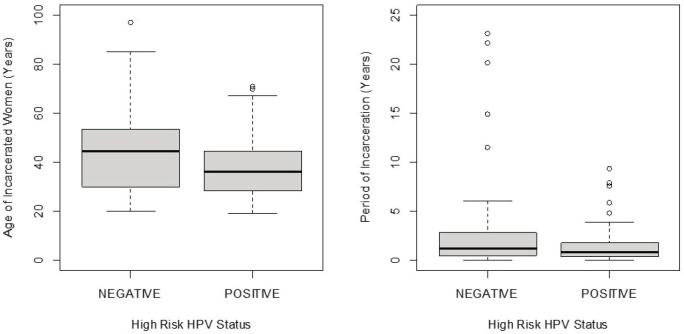
Box plot of the distribution of age and period of incarceration by hr-HPV status of incarcerated women.

Women with hr-HPV infection were younger (mean age = 37.8 years) than those without (mean age = 44.0 years); however, this difference was not statistically significant (*t*-test *p*-value = 0.0686). Similarly, women with hr-HPV infection had spent less time (median period of incarceration = 0.80 years) in prison than women without hr-HPV infection (median period of incarceration = 1.25 years); however, the difference was not statistically significant (Wilcoxon rank-sum test *p*-value = 0.2845). The distributions of age and period of incarceration for incarcerated women are shown in Figure 2.

## Discussion

Cervical cancer screening in LMICs has lagged behind that of high-income countries. This accounts for the higher rates of cervical cancer and cervical cancer deaths in LMICs. The introduction and adoption of primary HPV testing as the preferred mode for screening for cervical cancer has brought to the fore the possibility of same day see and treat protocols, increasing screening intervals and relying on self-sampling to improve cervical cancer screening in LMICs [19].

The prevalence rate of HIV among women in Ghana aged 15–49 years according to the Joint United Nations Programme on HIV/AIDS estimates is 2.3% [20]. The self-reported HIV positivity rate for the incarcerated women was 13.1% (95% CI: 7.5–21.9), which is lower than the rate of 15.6% (95% CI: 11.8–19.8) reported among prisoners in East and South Africa but higher than the rate of 8.2% (95% CI: 6.2–10.5) reported among incarcerated persons in West and Central Africa [21, 22]. These differences are not statistically significant though since their CIs overlap. All women who declared being HIV positive were receiving antiretroviral therapy. The HPV positivity rate among the self-declared HIV positive women was 63.6% (95% CI: 35.4–84.8) [23]. The HPV positivity rate among the HIV negative women and those with unknown HIV status was 42.4% and 57.1%, respectively. Among HIV-negative and -positive women living in Kumasi, Ghana, the HPV prevalence rates were 42% and 76.6%, respectively [24]. The prevalence rate of hr-HPV infection among the women (47.6%) was higher than the rate of 10.7% reported among outpatient gynaecologic patients assessed in Accra [25], 13.9% among pregnant women living in the Western region of Ghana [26] and 32.3% among women living in the North Tongu District of the Volta Region of Ghana [27]. Our findings compare with those of Escobar and Plugge who report a prevalence ranging from 10.5% to 55.4% with significant heterogeneity in their meta-analysis. In line with their findings, they report a wide prevalence range of CIN diagnosed by cytology in prisoners ranging from 0% to 22%. Their reported ratios comparing the prevalence of CIN in incarcerated women to that in the community ranged from 1.13 to 5.46. In their estimation, prevalence rates of cancer were at least 100 times higher than in non-incarcerated populations participating in national programmes. Our sample size was small and did not allow such comparisons, though the high rate of HPV positivity suggests a similar trend [12].

**Figure 3.**
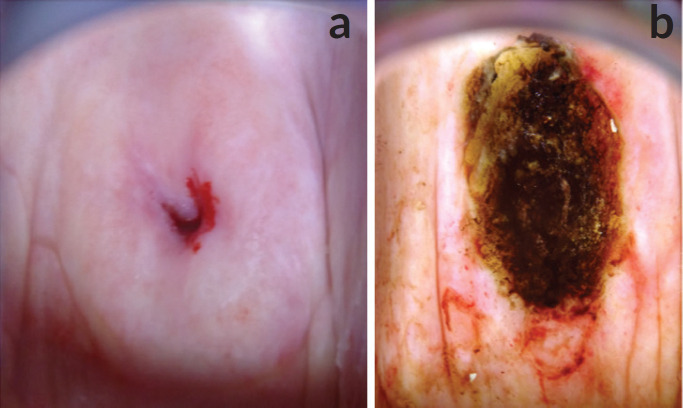
Colposcopy images of a 67-year-old incarcerated woman, para 7. HPV DNA testing-positive for high risk (others). Colposcopy: TZ3, leukoplakia at 12 O’clock entering the endocervical canal. Colposcopy: adequate, TZ3. Pap smear: atypical squamous cells, cannot exclude high grade intraepithelial lesion (ASC-H). Treatment: LEEP. Histopathology: No dysplasia. (a) Cervix, before application of acetic acid. (b) Cervix immediately after LEEP.

**Figure 4.**
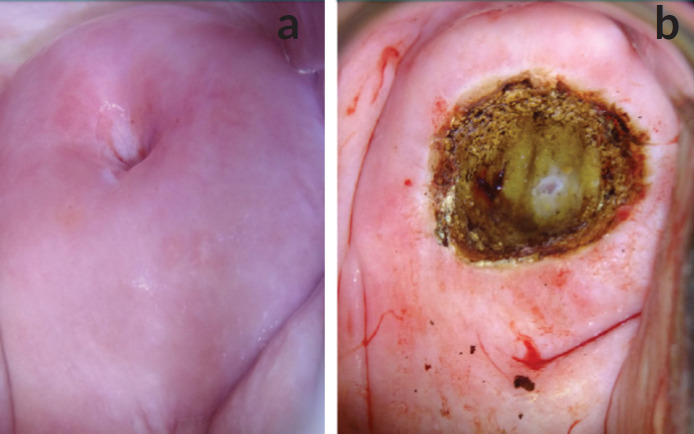
Colposcopy images of a 62-year-old incarcerated woman, para 4. HPV DNA testing-negative. Colposcopy: TZ3, leukoplakia at 11 O’clock extending into the endocervical canal. Pap smear: NILM. Treatment: LEEP. Histopathology: No dysplasia. (a) Cervix, before application of acetic acid. (b) Cervix immediately after LEEP.

The national policy on cervical cancer in Ghana recommends screening with VIA for women aged 25–45 years and cytology/Pap smear for women aged above 45years [28]. This is because the squamocolumnar junction generally recedes into the endocervical canal with age. This policy was developed before HPV DNA testing became readily available for clinical work in Ghana. The mean age of our study participants was 41.1 (standard deviation: 15.5) years. Thirty-five (35.7%) of the 84 incarcerated women were above 45 years old, which means VIA or screening with mobile colposcopy would not have been the recommended screening modality for them. 65.5% of the women had TZ3. This means the full circumference of the squamocolumnar junction was not visible (Figures 3 and 4). Using VIA or mobile colposcopy alone for screening would have been inadequate for these women as lesions in the endocervical canal could have been missed. Such women need to be screened with cytology or molecular tests (such as HPV DNA testing) to be able to detect possible endocervical lesions. This is in line with current WHO recommendations [29]. However, HPV DNA testing, despite its recommendation by the World Health Organization is difficult to adopt in low resource settings. The high sensitivity of HPV DNA testing compared with cytology leads to more screen positives that require triage and follow-up. In low resource settings with inadequate number of medical doctors and specialists to perform colposcopy, overtime, this can overwhelm health systems [20, 29]. Our approach of using the novel AmpFire HPV detection system which can give results in less than 3 hours concurrently with colposcopy by trained nurses using a mobile colposcope ensures that patients who test positive are immediately triaged by colposcopy, ensuring that those requiring follow-up (treatment) are appropriately managed. In Catholic Hospital, Battor, six nurses have been trained to perform colposcopy using the mobile colposcope. A gynaecologist reviews images for unclear cases. A monthly Quality Assurance meeting ensures that all management options for positive cases are reviewed by a gynaecologist. Based on TZ type, VIA/colposcopy was appropriate for primary screening for 34.5% of the women. Of these women, 13.1% had TZ1 (squamocolumnar junction fully ectocervical) and 21.4% had TZ2 (squamocolumnar junction partly or fully in the endocervical canal but the full circumference was fully visible). This information is important for screening programmes that would like to use visual inspection methods (because they are cheaper or easier to perform) as lesions may be missed when the full TZ is not visible.

**Figure 5.**
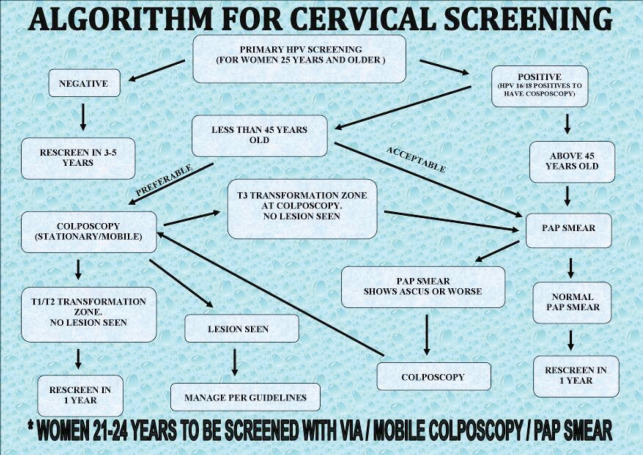
Algorithm for cervical cancer screening developed by the CCPTC, Battor.

**Figure 6.**
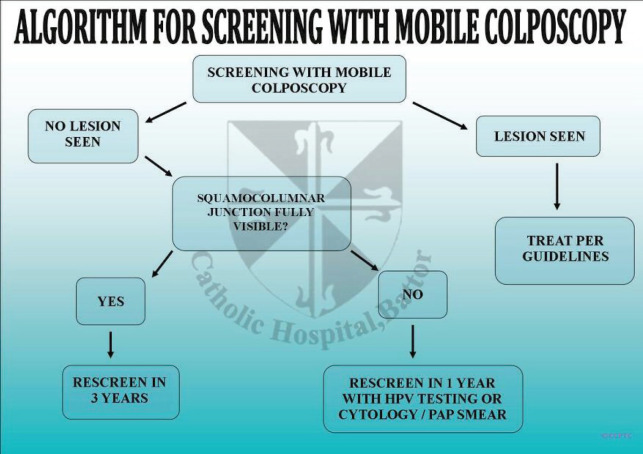
Algorithm for cervical cancer screening using the mobile colposcope.

**Figure 7.**
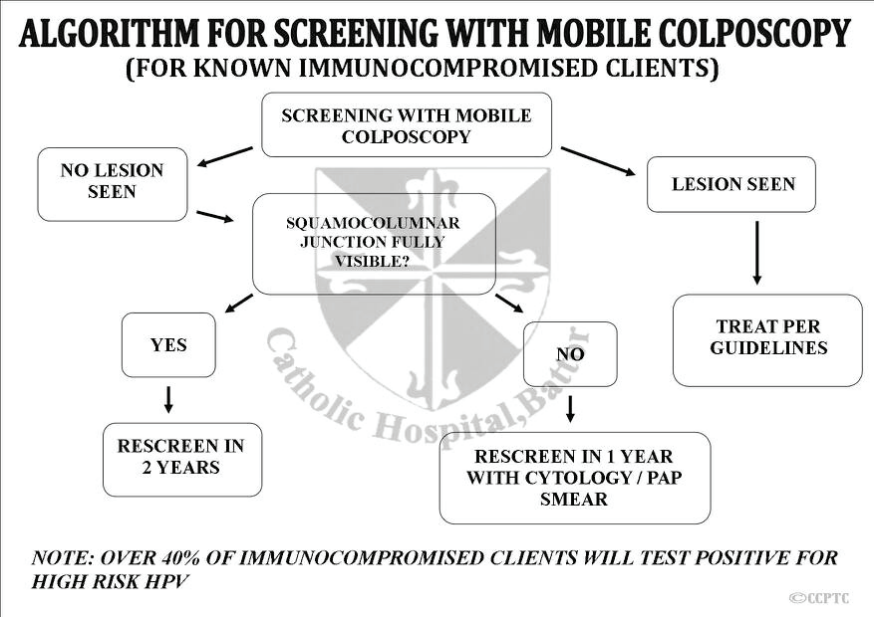
Algorithm for cervical cancer screening with the mobile colposcope for known HIV positive patients.

Women who tested positive on screening were followed up using algorithms developed by and being used by the CCPTC at Catholic Hospital, Battor (Figures 5–7). We recommend Pap smears after a year for women who test hr-HPV positive with TZ3 with no lesions on the ectocervix. If the women are available and can afford it or resources are available, the Pap smear can be taken after a month. Pap smears are not taken for these women at the initial screening because of low cell yield after taking a sample for HPV DNA testing and cleaning the cervix with acetic acid during VIA or colposcopy.

Twenty-one out of the 40 women who tested positive for hr-HPV (52.5%) had TZ3 (entire circumference of the squamocolumnar junction not visible). The management per our protocol (Figure 5) is follow-up with Pap smears to rule out a high-grade lesion in the endocervical canal. Nineteen out of the 21 availed themselves for Pap smears 3 weeks after the initial screening. One of the Pap smears was reported as atypical squamous cells, cannot exclude high grade intraepithelial lesion (ASC-H) (Figure 3). The other 18 Pap smear test results were negative for intraepithelial lesion or malignancy (NILM).

**Figure 8.**
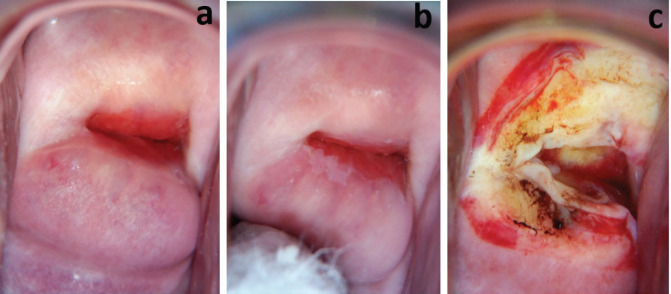
Colposcopy images of a 42-year-old incarcerated woman, para 2. HPV DNA testing-positive (for others). Colposcopy: TZ1, minor change (a): before acetic and (b): after acetic acid). (c): Treatment: thermal coagulation.

Five women were treated. Three of them were treated by thermal coagulation on the same day of screening (Figures 8a–c), while two of them with lesions not amenable to ablation were treated with LEEP under local anaesthesia in the Prison theatre some weeks after their Pap smears (Figures 3a,and b, and Figure 4a, and b).

Tables 1 and 2 show the categories of prisoners and the numbers and percentages of each category. Just over half (11 out of 21) of the women who had served for more than 3 years tested positive for hr-HPV. None of the four life prisoners but 1 (20.0%) out of the 4 condemned prisoners tested positive for hr-HPV. The last execution of condemned prisoners was carried out on the night of 17th July 1993 [30]. This means that condemned prisoners who are hr-HPV positive remain at risk of cervical cancer. The high prevalence of hr-HPV infection among the incarcerated women (47.6%) coupled with the absence of an organised cervical cancer screening programme across Ghana including the prisons means there is a risk of HPV infection persistence and progression to cervical cancer among many prisoners. This calls for an organised cervical cancer screening programme for incarcerated women, an at-risk population, that may not have the freedom to seek cervical cancer prevention services on their own.

^a^Remand prisoner: A person confined in prison whose case is awaiting hearing or pronouncement of sentence in a lower court (community tribunal, magistrate court or circuit court). Life prisoner: A person sentenced to spend the whole of his or her life in prison custody by a court of competent jurisdiction. Condemned prisoner: Any person who is found guilty by the court of justice and sentenced to death by hanging or firing squad by a court of competent jurisdiction. Convict prisoner: A person who has committed a crime and has been tried and found guilty by a court of competent jurisdiction and has been sentenced to serve a specific term in prison.

## Limitations

This work was not done in the context of a research study. The incarcerated women were offered the same services as clients of the CCPTC at Catholic Hospital, Battor. The same prescreening forms were filled before the incarcerated women were screened. Therefore, questions like history of intravenous drug abuse, sharing needles, illicit drug use, exchanging sex for drugs or money, among others, which are all risk factors for acquiring HIV and predisposing incarcerated women to hr-HPV infection, were not directly asked. The AmpFire HPV detection system does not offer full genotyping and lumps 13 hr-HPV infections together. It was, therefore, impossible to assess the most common hr-HPV types among the incarcerated women, and also impossible to detect multiple HPV infections especially when the multiple infections were due to hr-HPV types other than HPV 16 and 18. Also, it is likely that the true incidence of HIV infection among the incarcerated women is higher than documented here as the figure presented here was based on self-report among incarcerated women with no onsite HIV testing performed.

## Conclusions

We present the first results of the prevalence of hr-HPV among incarcerated women in Ghana. With this high prevalence of hr-HPV (47.6%), and 13.1% having been in prison for at least 5 years, many incarcerated women are at an elevated risk of developing cervical cancer if cervical cancer prevention services are not offered in the prisons.

We have demonstrated that it is possible to offer a high level of onsite screening and treatment of precancerous lesions of the cervix even for incarcerated women in a prison using current technologies such as HPV DNA testing by the AmpFire HPV detection system (Atila Biosystems, Mountain View, CA, USA), mobile colposcopy with the EVA system (MobileODT, Israel) and thermal coagulation and LEEP with the Cure/Liger Medical thermal coagulator and electrosurgical unit (Liger/Cure Medical LLC, Utah, USA).

## Conflicts of interest

None.

## Authors’ contributions

Conceptualisation: KE, ET and KAL. Participant screening, treatment and data collection: ET, CMW, IG, SK, ACK, PAD, NOME, MA, DA, KAL and KE. Data collation: ET, CMW, IG, SK, ACK, PAD, NOME, MA and DA. Formal analysis: KE and JEA. Writing – original draft: NOME, PKA, JEA, KAL and KE. All the authors read and approved the manuscript in its current form.

## Figures and Tables

**Table 1.** Sociodemographic and clinical characteristics of incarcerated women who underwent cervical screening at the Nsawam Medium Security Prison on 27–28 September 2019.

Characteristics of incarcerated women	Estimate
Age, mean (standard deviation)	41.1 (15.5)
Period of incarceration (years), median (IQR)	0.99 (0.47, 3.0)
Incarcerated for at least 5 years	13.1 (11)
Sentence type^a^, % (*n*) Remand Convict Lifers Condemn	14.2 (12) 75.0 (63) 4.8 (4) 6.0 (5)
Period of sentence (years), median (IQR) (denominator = 63)	5.0 (2.0, 10.0)
Marital status, % (*n*) Single Has a steady partner Married Divorced Widowed	19.1 (15) 15.5 (14) 25.0 (21) 22.5 (19) 17.9 (15)
Number of children 0 1 2 3+	17.9 (15) 21.4 (18) 14.3 (12) 46.4 (36)
Highest level of education, % (*n*) No formal education Elementary education Secondary education Tertiary education	17.9 (15) 15.5 (13) 52.4 (44) 14.3 (12)
Religious faith, % (*n*) Christian Islam African traditional religion	84.5 (71) 13.1 (11) 2.4 (2)
Smoker, % (*n*)	10.7 (9)
HIV positive, % (*n*)	13.1 (11)
Previous HPV vaccination, % (*n*)	1.2 (1)
Adequate view with mobile colposcopy, % (n)	100.0 (84)
TZ types, % (*n*) TZ1 TZ2 TZ3	13.1 (11) 21.4 (18) 65.5 (55)
Cervical lesions, % (*n*)	6.0 (5)
Treated for cervical lesions, % (*n*) Thermal coagulation LEEP	3.6 (3) 2.4 (2)
hr-HPV positive, % (*n*)	47.6 (40)
hr-HPV types, % (*n*) HPV 16 only HPV 18 only Other HPV types only HPV 16 and other HPV types HPV 18 and other HPV types	4.8 (4) 9.5 (8) 27.4 (23) 2.4 (2) 3.6 (3)
hr-HPV among HIV+ incarcerated women, % (*n*) (denominator = 11)	63.6 (7)

**Table 2.** Prevalence of hr-HPV by period of incarceration and prisoner category.

	Number of hr-HPV positive incarcerated women	Number of incarcerated women	Prevalence rate of hr-HPV (%)
Period of incarceration (years)			
<1	21	40	52.5
1–3	8	23	34.8
>3	11	21	52.4
Prisoner category			
Condemned	1	5	20.0
Life	0	4	0.0
Convict	32	63	50.8
Remand	7	12	58.3
